# Genetic Variants in DNA Repair Pathways as Potential Biomarkers in Predicting Treatment Outcome of Intraperitoneal Chemotherapy in Patients With Colorectal Peritoneal Metastasis: A Systematic Review

**DOI:** 10.3389/fphar.2020.577968

**Published:** 2020-10-06

**Authors:** Emma C. Hulshof, Lifani Lim, Ignace H. J. T. de Hingh, Hans Gelderblom, Henk-Jan Guchelaar, Maarten J. Deenen

**Affiliations:** ^1^ Department of Clinical Pharmacy, Catharina Hospital, Eindhoven, Netherlands; ^2^ Department of Clinical Pharmacy and Toxicology, Leiden University Medical Center, Leiden, Netherlands; ^3^ Department of Surgical Oncology, Catharina Hospital, Eindhoven, Netherlands; ^4^ GROW, School for Oncology and Development Biology, Maastricht University, Maastricht, Netherlands; ^5^ Department of Medical Oncology, Leiden University Medical Center, Leiden, Netherlands; ^6^ Leiden Network for Personalized Therapeutics, Leiden, Netherlands

**Keywords:** biomarker, colorectal cancer, DNA repair, hyperthermic intraperitoneal chemotherapy, mitomycin C, oxaliplatin, treatment outcome

## Abstract

**Background:**

The introduction of cytoreductive surgery (CRS) followed by hyperthermic intraperitoneal chemotherapy (HIPEC) with either oxaliplatin or mitomycin C for patients with colorectal peritoneal metastasis (CPM) has resulted in a major increase in overall survival. Nonetheless, despite critical patient selection, the majority of patients will develop recurrent disease within one year following CRS + HIPEC. Therefore, improvement of patient and treatment selection is needed and may be achieved by the incorporation of genetic biomarkers. This systematic review aims to provide an overview of genetic biomarkers in the DNA repair pathway that are potentially predictive for treatment outcome of patients with colorectal peritoneal metastases treated with CRS + HIPEC with oxaliplatin or mitomycin C.

**Methods:**

A systematic review was conducted according to the PRISMA guidelines. Given the limited number of genetic association studies of intraperitoneal mitomycin C and oxaliplatin in patients with CPM, we expanded the review and extrapolated the data from biomarker studies conducted in colorectal cancer patients treated with systemic mitomycin C– and oxaliplatin-based chemotherapy.

**Results:**

In total, 43 papers were included in this review. No study reported potential pharmacogenomic biomarkers in patients with colorectal cancer undergoing mitomycin C–based chemotherapy. For oxaliplatin-based chemotherapy, a total of 26 genetic biomarkers within 14 genes were identified that were signiﬁcantly associated with treatment outcome. The most promising genetic biomarkers were *ERCC1* rs11615, *XPC* rs1043953, *XPD* rs13181, *XPG* rs17655, *MNAT* rs3783819/rs973063/rs4151330, MMR status, ATM protein expression, *HIC1* tandem repeat D17S5, and *PIN1* rs2233678.

**Conclusion:**

Several genetic biomarkers have proven predictive value for the treatment outcome of systemically administered oxaliplatin. By extrapolation, these genetic biomarkers may also be predictive for the efficacy of intraperitoneal oxaliplatin. This should be the subject of further investigation.

## Introduction

Colorectal peritoneal metastasis (CPM) is associated with a poor prognosis and affects approximately 10–20% of colorectal cancer patients (Chu et al., 1989; Jayne et al., 2002; Verwaal et al., 2003; Lemmens et al., 2011). The introduction of cytoreductive surgery (CRS) followed by hyperthermic intraperitoneal chemotherapy (HIPEC) with either oxaliplatin or mitomycin C for patients with isolated CPM has led to a major increase in overall survival and even cure in up to 15% of patients (Sugarbaker, 1995; Goere et al., 2013). Therefore, CRS + HIPEC is at present considered standard of care for patients with limited peritoneal metastases. Currently, patient selection for CRS + HIPEC is mainly based on the peritoneal carcinomatosis index (PCI) and performance status (Froysnes et al., 2016; Kwakman et al., 2016; Kusamura et al., 2016). In addition, several clinical and pathological prognostic biomarkers have been identified, including completeness of cytoreduction, locoregional lymph node status and signet ring cell differentiation (Simkens et al., 2017). Nonetheless, despite critical patient selection, the majority of patients will develop recurrent disease within one year following CRS + HIPEC (Konigsrainer et al., 2013; Braam et al., 2014). In addition, post-operative surgical complications following CRS + HIPEC are frequent, including mortality in about 1–2% of patients (Chua et al., 2009).

Knowledge of genetic biomarkers that are predictive or prognostic for treatment outcome may be of additional value in patient and treatment selection, allowing further improvement of treatment outcome for the individual patient. In contrast to thousands of pharmacogenetic association studies that have been conducted in cancer patients treated with systemic chemotherapy, almost no data exist of genetic biomarkers in patients treated with intraperitoneal chemotherapy. Following intraperitoneal administration, oxaliplatin and mitomycin exert their anti-tumor effect locally at the tumor site. Both drugs share a comparable mechanism of action in that they both interfere with DNA synthesis and repair. Thereby, genetic variation in genes involved in DNA repair may reduce the functional activity of certain DNA repair genes, making tumor cells more susceptible for drug-induced DNA damage and hence increased drug efficacy (Kweekel et al., 2005; D’Andrea, 2014). The DNA repair system is divided into six major DNA repair pathways, i.e. base-excision repair (BER), nucleotide-excision repair (NER), mismatch repair (MMR), homologous recombination (HR), nonhomologous end joining (NHEJ), and translesion DNA synthesis (TLS). In addition, pathways on damage response and DNA synthesis exist (D’Andrea, 2014).

Notwithstanding the in general increasingly applied knowledge of genetic biomarkers in cancer therapy as a proven tool for patient and treatment selection, almost no predictive or prognostic data of genetic biomarkers for treatment outcome exist in patients with CPM treated with intraperitoneal chemotherapy. Therefore, we conducted a systematic review to provide an overview of genetic biomarkers in the DNA repair pathway that are potentially predictive for treatment outcome of patients with colorectal peritoneal metastases treated with CRS + HIPEC with oxaliplatin or mitomycin C.

## Methods

A systematic literature review was conducted according to the Preferred Reporting Items for Systematic Reviews and Meta-Analyses (PRISMA) guidelines (Moher et al., 2009).

Of the studies on the use of mitomycin C and oxaliplatin in HIPEC treatment, only two studies were found that have reported biomarkers related to DNA repair (Massalou et al., 2017; Shannon et al., 2019). Data obtained from genetic association studies conducted in other than CPM patients treated with oxaliplatin or mitomycin C may potentially be extrapolated to patients with CPM. Therefore, we expanded this review with studies investigating the association between genetic biomarkers related to DNA repair and treatment outcome in patients with colorectal cancer undergoing mitomycin C– and oxaliplatin-based chemotherapy.

We searched PubMed until February 2020 without any limitations on publication year using the following search terms: “biomarker,” “oxaliplatin,” “mitomycin C,” “colorectal cancer,” and “treatment outcome.” The full search string is provided in the **Supplementary Material**. In addition, reference lists in original articles and review articles were manually searched to identify additional potentially relevant publications. Literature was reviewed by two independent reviewers (LL and EH). In case of inconsistencies, results were discussed with a third reviewer (MD).

All publications were screened on title and abstract. Only studies that included patients with colorectal cancer were included, and studies that were retracted and studies that did not provide original data or case reports were excluded. The remaining publications were assessed based on screening of the full text. Only studies that reported on the association between genetic biomarkers related to DNA repair and treatment outcome undergoing mitomycin C– and oxaliplatin-based chemotherapy were included. To provide a total overview of the available evidence, we included studies on various types of genetic biomarkers including genetic polymorphism, mRNA expression, and protein expression. Treatment outcome had to be reported as overall survival (OS), progression-free survival (PFS), or disease-free survival (DFS).

Risk of bias assessment was performed and adapted from the Q-genie tool and was based on the following bias items: clear phenotype and outcome definition and correct nomenclature of genotype. We decided not to exclude studies because of small sample size, ethnic differences, differences in treatment regimens or type of biomarker, or no correction for covariates affecting treatment outcome due to scarcity of data.

All identified genetic biomarkers were subdivided into either one of the six described major DNA-repair pathways (Mendelsohn et al., 2015), i.e., NER, BER, MMR, HR, NHEJ, TLS, or otherwise into a category of genes involved in DNA damage response and DNA synthesis (D’Andrea, 2014). Results were summarized and presented per gene including a mechanistic background for the drug-gene interaction. The following information per study or genetic biomarker was reported: sample size, CRC type, treatment schedule, biomarker, type of sample, type of assay, rs number (if applicable), reference group and comparator group, and treatment outcome. Treatment outcomes were expressed as hazard ratios, relative risks, or differences in median survival with 95% confidence intervals and p-values, whichever was available.

The most promising genetic biomarkers were extracted from the results and summarized in a table. Evidence for these biomarkers had to meet the following 2 criteria: (1) no or almost none conflicting data and (2) an association with treatment outcome was reported in at least two studies or in one study with sufficient power (arbitrarily defined in this review as a minimum number of 300 patients) or the study included a control group with non-oxaliplatin based–chemotherapy in which no association or an association in the opposite direction was seen compared to the group with oxaliplatin-based chemotherapy.

## Results

### Study Selection

**Figure 1:**
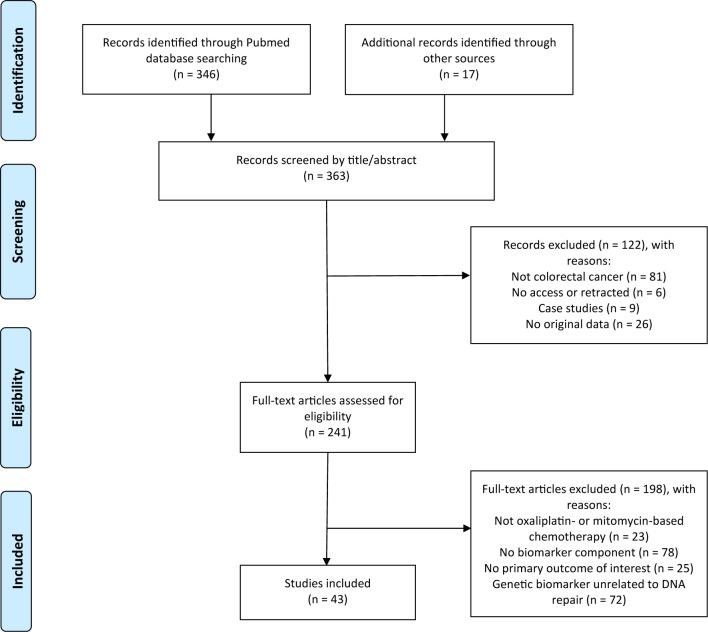
Flow chart of selection procedure literature.

The search string in the PubMed database resulted in a total of 346 identified articles. **Figure 1** provides the selection procedure of relevant articles. An additional 17 studies were added that were identified from meta-analyses. After screening the title and abstract, 122 studies were excluded leaving 241 articles for further evaluation. After reviewing the full-text, 198 articles were excluded, resulting in a total of 43 studies that were included in this systematic review. The percent agreement between the two reviewers was 97%, and Cohen’s kappa was 0.87.

### Main Results

**Figure 2:**
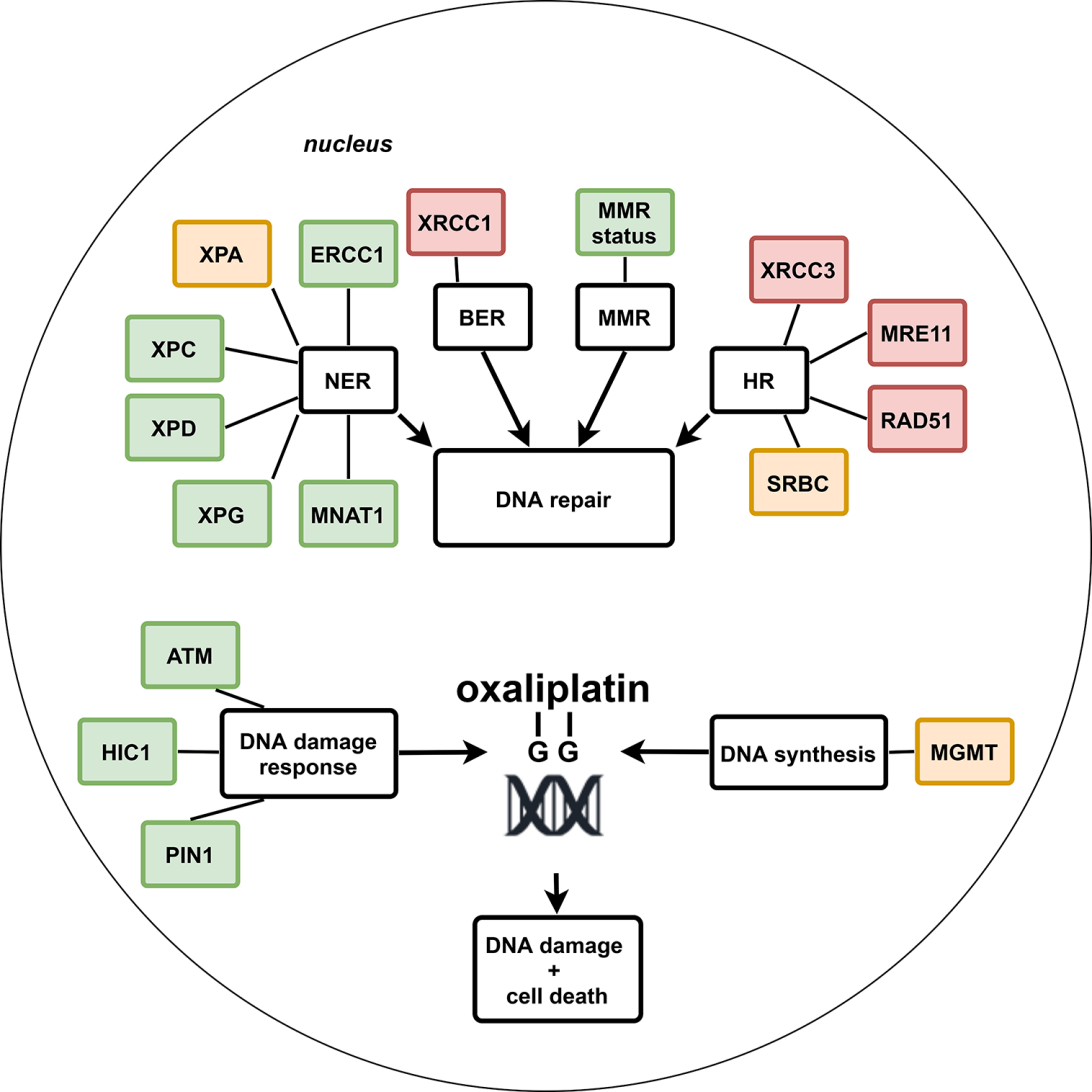
Schematic overview of potential genetic biomarkers within DNA repair pathways for treatment outcome of systemic oxaliplatin in colorectal cancer patients. Green: no or almost none conflicting results and significant association with treatment outcome in ≥2 studies, or in one study with a sample size of ≥300, or inclusion of a non-oxaliplatin–based chemotherapy control group in which no association or an association in the opposite direction was seen compared to the group with oxaliplatin-based chemotherapy. Orange: significant association with treatment outcome in one study. Red: conflicting results or no significant association with treatment outcome.

The identified potential genetic biomarkers for treatment outcome of oxaliplatin-based chemotherapy could be divided over four out of the six major DNA-repair pathways, i.e., NER, BER, MMR, and HR or were involved in DNA damage response or DNA synthesis, respectively. No studies were identified that reported on the association between genetic biomarkers and treatment outcome of mitomycin C–based chemotherapy in CRC patients. From all eligible studies, a total of 26 genetic biomarkers within 14 genes were identified in which at least one study had reported a signiﬁcant association with treatment outcome. The most promising genetic biomarkers belonged to the NER, MMR, or DNA damage response pathway and are summarized in **Table 1** and explained in more detail below; in contrast to biomarkers that belong to the BER, HR, or DNA synthesis pathway, which seem less promising due to lack of evidence or conflicting results. The results from all included studies are summarized in **Figure 2**, discussed per gene below, and reported in detail in the **Supplementary Material—Tables S1–S10**.

**Table 1:** Overview of most promising genetic biomarkers within DNA repair for treatment outcome of hyperthermic intraperitoneal chemotherapy in colorectal cancer patients.

Biomarker	Location	Pathway	Favorable genotype/expression
*ERCC1 c.354T>C^A^*	rs11615	NER	CC
XPC c.*463A>G	rs1043953	NER	GG
*XPD c.2251A>C^B^*	rs13181	NER	AA
*XPG c.3310G>C*	rs17655	NER	GG
*MNAT1 c.688-30168A>G^C^*	rs3783819	NER	GG
*MNAT1 c.562-88A>G^C^*	rs973063	NER	GG
*MNAT1 c.809+24992A>G^C^*	rs4151330	NER	GG
MMR status	n.a.	MMR	MMR deficient
ATM protein expression	n.a.	DNA damage response	Loss of ATM expression
*HIC1* tandem repeat	D17S5	DNA damage response	≤4 tandem repeats
*PIN1 NC_000019.9:g.9945179G>C*	rs2233678	DNA damage response	GG

A Six studies reported that the CC genotype was favorable, and three studies reported that the TT genotype was favorable.

B Eight studies reported that the AA genotype was favorable, three studies reported that the CC genotype was favorable.

C SNPs are in high linkage disequilibrium.

#### NER Pathway

##### ERCC1

Oxaliplatin DNA adducts are mainly removed by the NER pathway (Shirota et al., 2001). Excision repair cross-complementation group 1 (ERCC1) is a key protein in the NER pathway that is encoded by the *ERCC1* gene. Together with xeroderma pigmentosum complementation group F (XPF), ERCC1 forms a heterodimer complex that can incise damaged DNA strands at the 5’ side of the lesion (Sijbers et al., 1996). In addition to their involvement in the NER pathway, the XPF/ERCC1 complex is also involved in double strand break repair (DSBR) (Ahmad et al., 2008). Therefore, the expression of *ERCC1* is potentially associated with treatment outcome of oxaliplatin in CRC patients.

In two preclinical studies, elevated ERCC1 protein level was suggested to correlate with oxaliplatin-resistance in cells (Boyer et al., 2004; Lin et al., 2012). Alteration in single nucleotide polymorphisms (SNPs) is expected to have an effect in gene expression level and function. Several *ERCC1* SNPs have been evaluated for their association with treatment outcome of oxaliplatin in CRC patients (**Supplementary Material—Table S1**). The most commonly investigated nucleotide polymorphism is rs11615 (Stoehlmacher et al., 2004; Ruzzo et al., 2007; Liang et al., 2008; Martinez-Balibrea et al., 2008; Pare et al., 2008; Chang et al., 2009; Chua et al., 2009; Chen et al., 2010; Liang et al., 2010; Huang et al., 2011; Lamas et al., 2011; Farina Sarasqueta et al., 2011; Li et al., 2012; Kumamoto et al., 2013; Nishina et al., 2013; van Huis-Tanja et al., 2014; Zaanan et al., 2014; Rao et al., 2019). A total of 10 studies showed a significant association between this polymorphism and treatment outcome (Stoehlmacher et al., 2004; Ruzzo et al., 2007; Martinez-Balibrea et al., 2008; Pare et al., 2008; Chang et al., 2009; Chua et al., 2009; Chen et al., 2010; Huang et al., 2011; Li et al., 2012; Rao et al., 2019). Most studies, six out of 10, reported the mutant CC genotype to be the favorable genotype, with significantly better DFS, PFS, and OS (Stoehlmacher et al., 2004; Chang et al., 2009; Chua et al., 2009; Chen et al., 2010; Huang et al., 2011; Li et al., 2012). However, a few studies showed contradictory results. Three studies (Martinez-Balibrea et al., 2008; Pare et al., 2008; Rao et al., 2019) reported that patients with the CC genotype had a worse treatment outcome in terms of PFS and OS. Another contradicting result was reported by Ruzzo et al. (2007) where the rs11615 TT genotype was associated with prolonged PFS in univariate analysis and shorter PFS in multivariate analyses.

Two other reported polymorphisms of *ERCC1* are at codon 259 and 504 (Monzo et al., 2007; Nishina et al., 2013). Both polymorphisms showed no significant association with treatment outcome. Moreover, two (Kassem et al., 2017; Rao et al., 2019) out of five (Basso et al., 2013; Li et al., 2014; Sfakianaki et al., 2019) studies based on mRNA or protein expression level of ERCC1 showed a significant association between low ERCC1 expression and prolonged PFS and OS.

##### XPC

*Xeroderma pigmentosum group C* (*XPC*), located at chromosome 3p25, encodes for another important protein in the early steps of the NER pathway. XPC binds to RAD23B to form the heterodimeric complex, which is the first NER factor to facilitate the recognition of DNA damage and the initiation of DNA repair (Sugasawa et al., 1998). As DNA damage recognition is the rate-limiting step in the NER pathway, the XPC protein plays a critical role in proper DNA repair. Therefore, genetic biomarkers in *XPC* may have potential value in predicting response for oxaliplatin-based chemotherapy.

In **Table S3** (**Supplementary Material**), three SNPs in the *XPC* gene that are potentially predictive of treatment response to oxaliplatin-based therapy in CRC patients are reported (Liu et al., 2012; Kap et al., 2015; Hu et al., 2019). Only one SNP was significantly associated with survival. In the study by Kap et al. (2015), patients carrying the variant allele rs1043953 had a longer OS after treatment with oxaliplatin-based chemotherapy compared to non-carriers after adjusting for multiple testing, while the opposite association was found in patients who were treated with non-oxaliplatin based–chemotherapy.

##### XPD/ERCC2

*The xeroderma pigmentosum group D* (*XPD*), or *excision repair cross complementation group 2* (*ERCC2*) gene, encodes for a helicase protein of 761 amino acids located on chromosome 19q13.3 (Weber et al., 1990). The XPD protein is a part of the general transcription factor IIH complex, which is involved in the NER pathway by opening DNA double helix after damage recognition by XPC-RAD23B (Oksenych and Coin, 2010). SNPs in *XPD* gene can alter the efficiency of DNA repair capacity and could thus be used as a predictive factor for oxaliplatin-based chemotherapy.

SNPs affecting codons 156, 312, and 751 (rs238406, rs1799793, and rs13181, respectively) proved to be extensively studied for their predictive value in CRC treatment (**Supplementary Material—Table S4**). *XPD* rs238406 SNP was significantly associated with treatment outcome in one (Kjersem et al., 2016) out of three studies (Park et al., 2001; Stoehlmacher et al., 2004). The second SNP, rs1799793, was also significantly associated with treatment outcome in one (Liu et al., 2019) out of three studies (Park et al., 2001; Ruzzo et al., 2007). The wild type GG genotype seemed to be the favorable genotype. Sixteen studies assessed the predictive value of *XPD* rs13181polymorphism. In most studies a worse treatment outcome was observed in C allele carriers (Park et al., 2001; Stoehlmacher et al., 2004; Le Morvan et al., 2007; Ruzzo et al., 2007; Pare et al., 2008; Lai et al., 2009; Chen et al., 2010; Kumamoto et al., 2013). Le Morvan et al. compared oxaliplatin treatment with irinotecan treatment and reported that the CC genotype was associated with a lower OS in patients treated with oxaliplatin, in contrast this was not observed in the same patient category treated with irinotecan (Le Morvan et al., 2007). However, the opposite association was observed in three studies (Lamas et al., 2011; Gan et al., 2012; Li et al., 2012), and five studies did not find significant associations with treatment outcome (Monzo et al., 2007; Martinez-Balibrea et al., 2008; Etienne-Grimaldi et al., 2010; Farina Sarasqueta et al., 2011; Huang et al., 2011). Lastly, one study assessed mRNA expression level of *XPD* for its association with treatment outcome, and no significant association was observed (Kassem et al., 2017).

##### XPG/ERCC5

The *xeroderma pigmentosum group G* (*XPG*) gene, also known as *ERCC5* (*excision repair cross complementation group 5*), is one of the eight core functional genes in the NER pathway. The *XPG* gene, located at chromosome 13q32-33, encodes for a structure specific endonuclease protein that cleaves the 3’ side of the damaged nucleotide during NER (Aboussekhra et al., 1995). The low expression level of *XPG* has been shown to be associated with response to platinum-based chemotherapy in ovarian cancer (Stevens et al., 2008; Walsh et al., 2008).

Four studies reported on the association between four different SNPs in the *XPG* gene and treatment outcome of oxaliplatin-based chemotherapy in CRC patients (**Supplementary Material—Table S5**). The -763A>G and +25A>G polymorphisms in the promoter region of *ERCC5* were significantly associated with PFS and OS in patients treated with oxaliplatin (Chen et al., 2016). Also, SNPs in rs1047768 and rs17655 were significantly associated with treatment outcome (Monzo et al., 2007; Kweekel et al., 2009; Liu et al., 2012).

##### MNAT1

The *MNAT1* gene encodes for the ménage à trois-1 (MAT1) enzyme that is involved in the assembly of the cyclin dependent kinase-activating kinase (CAK) complex. Together with XPD and other subunits, the CAK-complex forms the TFIIH complex that is involved in the NER pathway (Marinoni et al., 1997).

Kap et al. (2015) found three predictive SNPs, rs3783819, rs973063, and rs4151330 of the *MNAT1* gene for OS in CRC patients treated with oxaliplatin-based chemotherapy compared to CRC patients with non-oxaliplatin–based chemotherapy (**Supplementary Material—Table S6**). All three SNPs are in high linkage disequilibrium, and p-values were corrected for multiple testing. Compared to non-carriers, carriership of these genetic variants was associated with longer OS, but not in patients who received non-oxaliplatin–based chemotherapy.

#### MMR Pathway

##### MMR Status

The DNA mismatch repair (MMR) system recognizes and repairs genetic mismatches that occur during DNA replication and DNA damage. MMR status is defined as deficient (dMMR) when one or more MMR protein (*MLH1*, *MSH2*, *PMS2*, and *MSH6*) expression is lost (Ionov et al., 1993). Germline mutations in MMR genes were found to be the driving mechanism for Lynch syndrome, also known as hereditary nonpolyposis colorectal cancer (HNPCC) (Pino et al., 2009). A defective MMR system will result in DNA replication errors, particularly in the short tandem repeat of DNA sequences of the genome referred to as microsatellites, which may lead to microsatellite instability (MSI). It has been suggested that MSI positively affects the clinical outcome of CRC. Mechanistically, oxaliplatin treatment is expected to be more effective in patients with defective MMR protein status as platinum adducts formed by oxaliplatin cannot be repaired.

A total of three studies, evaluating the predictive ability of MMR status in relation to oxaliplatin-based treatment, are included in **Table S9** (**Supplementary Material**). In two out of three studies, OS was significantly higher in multivariate analysis in dMMR patients treated with oxaliplatin-based therapy (Gallois et al., 2018; Sfakianaki et al., 2019). In contrast, Kim et al. did not find an association between dMMR and treatment outcome of oxaliplatin-based chemotherapy (Kim et al., 2010).

#### DNA Damage Response

##### ATM

Ataxia telangiectasia mutated (ATM) is a serine/threonine protein kinase that is recruited and activated by the MRN complex during DNA DSBR (Uziel et al., 2003). The activation of the *ATM* gene leads to the phosphorylation of several key proteins that mediates the effect of ATM protein on DNA repair, cell cycle arrest, or apoptosis (Shiloh, 2003). Loss of *ATM* in preclinical models seems to increase sensitivity to DNA damaging agents, including platinum-based chemotherapy and ATM inhibitors (Reaper et al., 2011).

Two studies reported a significant association of *ATM* with treatment outcome of oxaliplatin in CRC patients (**Supplementary Material—Table S10**) (Kweekel et al., 2009; Sundar et al., 2018). Sundar et al. (2018) reported that loss of ATM protein expression in CRC resulted in favorable OS when treated with first line oxaliplatin chemotherapy (49 vs. 32 months; HR: 2.52 [1.00–6.37]). It is important to note that loss of ATM expression did not result in favorable OS among patients treated with first line irinotecan-based therapy (24 vs. 33 months; HR: 0.72 [0.28–1.84]). In addition, the explorative study by Kweekel et al. (2009) found a significantly shorter PFS for homozygous carriers of the *ATM* rs1801516 SNP, for OS no differences were found.

##### HIC1

The hypermethylated in cancer 1 (HIC1) protein plays an important role in the DNA repair through its direct binding to the Sirtuin 1 (SIRT1) promoter, thereby suppressing its transcription. SIRT1 is a deacetylase of XPA protein, a component of the NER pathway (Fan and Luo, 2010). Since the variable number of tandem repeats near the promoter lesion of HIC1, which is associated with *HIC1* gene expression, there is a potential value of *HIC1* as a predictive biomarker for oxaliplatin efficacy.

In a study by Okazaki et al. (2017), shown in **Table S10** (**Supplementary Material**), patients treated with oxaliplatin-based chemotherapy with at least five tandem repeats of *HIC1*, in both alleles of the *HIC1* promoter region, had a significantly shorter PFS. In a control group who received irinotecan-based chemotherapy this difference in PFS was not seen. However, no significant association with OS was found.

##### PIN1

Peptidyl-prolyl cis/trans isomerase NIMA-interacting 1 (PIN1) is an enzyme encoded by the *PIN1* gene. It interacts with prominent DSBR factors and is involved in the regulation of HR and non-homologous end-joining (NHEJ) of DNA DSBR. Previous study showed that the overexpression of PIN1 suppresses HR and its depletion reduces NHEJ by promoting CtIP polyubiquitylation and subsequent proteasomal degradation (Steger et al., 2013).

A study by Suenaga et al. (2018), shown in **Table S10** (**Supplementary Material**), reported that genetic polymorphism in *PIN1* was associated with treatment outcome of oxaliplatin. Patients treated with oxaliplatin-based chemotherapy carrying the *PIN1* rs2233678 C allele had a shorter PFS and OS compared to wild type patients. For OS this was replicated in a validation cohort. In contrast, in a control group treated with non-oxaliplatin-based chemotherapy patients with a C allele had longer median PFS than wild type patients.

#### Miscellaneous

Following our selection criteria, for XPA in the NER pathway (Stoehlmacher et al., 2004; Monzo et al., 2007; Hu et al., 2019), SRBC in the HR pathway (Moutinho et al., 2014) and MGMT in the DNA synthesis pathway (Park et al., 2010) results remain inconclusive because the observed associations have not yet been replicated and the studies itself were relatively small (<300 patients).

For XRCC1 in the BER pathway a total of nine studies were identified that assessed the association between the *XRCC1* gene and treatment outcome of oxaliplatin-based chemotherapy in CRC patients, and showed conflicting results (Suh et al., 2006; Martinez-Balibrea et al., 2008; Chua et al., 2009; Liang et al., 2010; Huang et al., 2011; Lamas et al., 2011; Gan et al., 2012; Zaanan et al., 2014). All nine studies investigated the *1196A>G* polymorphism, and three studies showed a significant association (Suh et al., 2006; Huang et al., 2011; Gan et al., 2012). However, two out of three studies (Suh et al., 2006; Huang et al., 2011) found a significantly longer OS for the GG genotype, whereas the other study (Gan et al., 2012) a longer OS for the AA genotype.

For XRCC3 (Ruzzo et al., 2007; Martinez-Balibrea et al., 2008), MRE11 (Ihara et al., 2016), and RAD51 (Ihara et al., 2016) in the HR pathway, no significant associations with treatment outcome were reported.

## Discussion

The majority of patients with peritoneal metastases of colorectal cancer treated with CRS + HIPEC will develop recurrent disease despite critical patient selection. Therefore, improvement of patient and treatment selection is needed and further investigation of genetic biomarkers that are predictive or prognostic for treatment outcome may be of aid herein. We conducted a systematic review to provide an overview of genetic biomarkers in the DNA repair pathway that are potentially predictive for treatment outcome of patients with colorectal peritoneal metastases treated with CRS + HIPEC with oxaliplatin or mitomycin C.

We expanded our review with studies investigating the association between genetic biomarkers related to DNA repair and treatment outcome in patients with colorectal cancer undergoing systemic chemotherapy, because only two studies could be retrieved that investigated the association of biomarkers related to DNA repair and intraperitoneally administered mitomycin C or oxaliplatin. The most promising genetic biomarkers were *ERCC1* rs11615*, XPC* rs1043953*, XPD* rs13181*, XPG* rs17655*, MNAT* rs3783819/rs973063/rs4151330, MMR status, ATM protein expression, *HIC1* tandem repeat D17S5 and *PIN1* rs2233678. Combination studies of two DNA repair genes have also been studied and showed significant associations with treatment outcome.

Our findings for *ERCC1* rs11615 and *XPD* rs13181 are supported in four meta-analyses (Yin et al., 2011; Qian et al., 2014; Ma et al., 2015; Shahnam et al., 2016). The other biomarkers have not been studied as extensively. To our knowledge the current review is the first to summarize the available evidence for these markers.

Our results showed that genetic biomarkers in the DNA repair pathway seem of added value in predicting oxaliplatin treatment outcome in colorectal cancer patients. Since the mechanism of action of oxaliplatin is irrespective of the route of administration, it is assumed very reasonable to extrapolate these associations to patients with colorectal peritoneal metastases treated with CRS + HIPEC. In our opinion, single genetic biomarkers within DNA repair should be incorporated into a polygenic risk profile because the effect of a single gene polymorphism may be partially overcome by compensation mechanisms. Comparable to the study by Kap et al., in which the predictive value of the model significantly improved by including more genetic variants (Kap et al., 2015). Moreover, besides DNA repair, other pathways may also be of relevance in predicting treatment outcome, such as genetic variation in pharmacokinetic genes (Hulshof et al., 2020).

For some genetic biomarkers conflicting results were reported. This might partially be explained by ethnic discrepancy as has been suggested (Yin et al., 2011; Ma et al., 2015). In addition, studies with small sample sizes and differences in treatment regimens between studies may also attribute to these conflicting results. However, for the selection of the most promising genetic biomarkers, we only selected biomarkers for which no or almost none conflicting data existed and results had to be replicated in at least two studies or in one study with sufficient power (>300 patients) or the study had to have a control group with non-oxaliplatin based chemotherapy.

Moreover, genetic variants in the DNA repair pathway seem to affect cancer susceptibility, prognosis and treatment outcome (Dai et al., 2015). Therefore, it is difficult to distinguish between prognostic effects of these genetic variants or predictive effects on treatment outcome of oxaliplatin. To differentiate between these prognostic effects and predictive effects, a control group consisting of a patient cohort treated with non-oxaliplatin based chemotherapy should be added. Most of the studies that were included had no control group. Nonetheless, the studies that did include a control group with non-oxaliplatin based-chemotherapy did find differences in the association between the genetic biomarker (*XPC* rs1043953, *XPD* rs13181, *MNAT* rs3783819/rs973063/rs4151330, ATM protein expression, *HIC1* tandem repeat D17S5, and *PIN1* rs2233678) and treatment outcome of oxaliplatin-based chemotherapy and non-oxaliplatin based–chemotherapy, thereby suggesting these biomarkers to be more likely predictive than prognostic.

In addition, we included various types of biomarkers such as genetic polymorphism, mRNA expression and protein expression, these are quite different assays and normally we would not pile together these various types of biomarkers. However, our aim was to give a complete overview of all genetic biomarkers in order to provide a selection of potential promising genetic biomarkers for further research.

As data scarcity and sparsity were encountered, we decided to expand our search from intraperitoneal chemotherapy to systemic chemotherapy. No formal search in other databases than PubMed was conducted, since it was assumed that the majority of relevant literature was identified using this database. However, this might be considered a limitation of our study. Moreover, the addition of gray literature could have been of added value in terms of data scarcity and publication bias. Nonetheless, gray literature is mostly not peer-reviewed and not always traceable. In addition, the quality of data could potentially be improved by applying a standardized tool for the risk of bias assessment. However, as described in the methods section, a customized assessment of bias was performed which was mainly based on the Q-genie tool.

Lastly, not all studies corrected for additional covariates affecting treatment outcome such as clinical, molecular, and pathological patient and tumor characteristics. This might have influenced the effect of the genetic biomarkers on treatment outcome. Therefore, additional prospective research including a multivariate analysis is needed, especially in patients with colorectal peritoneal metastases treated with CRS + HIPEC as literature is scarce in this population.

In this review, several genetic biomarkers in the DNA repair pathway were identified that showed promise for predicting outcome in colorectal cancer patients treated with oxaliplatin. These findings might be extrapolated to patients with colorectal peritoneal metastases treated with CRS + HIPEC and should be the subject of further investigation.

## Data Availability Statement

The original contributions presented in the study are included in the article/**Supplementary Material**; further inquiries can be directed to the corresponding author.

## Author Contributions

Study design: EH and MD. Literature search, data interpretation, and data analysis: EH, LL, and MD. Manuscript writing: EH, LL, and MD, Critical revision of data presentation and manuscript: IH, HG, and HJG. Approval final version of manuscript: EH, LL, IH, HG, HJG, and MD.

## Conflict of Interest

The authors declare that the research was conducted in the absence of any commercial or financial relationships that could be construed as a potential conflict of interest.
