# Supplementary material for: Genetic Variants in DNA Repair Pathways as Potential Biomarkers in Predicting Treatment Outcome of Intraperitoneal Chemotherapy in Patients With Colorectal Peritoneal Metastasis: A Systematic Review
Source: Front Pharmacol. 2020 Oct 6;11:577968. doi: 10.3389/fphar.2020.577968 (PMC7575928; doi:10.3389/fphar.2020.577968)
Supplement: Supplementary file 1 [file DataSheet_1.docx]

Supplementary material belonging to the manuscript entitled:

Genetic Variants in DNA Repair Pathways as Potential Biomarkers in Predicting Treatment Outcome of Intraperitoneal Chemotherapy:

a Systematic Review

**E.C. Hulshof^1,2^, L. Lim^2^, I.H.J.T. de Hingh^3,4^, H. Gelderblom^5^, H.J. Guchelaar^2,6^, M.J. Deenen^1,2*^**

^1^Department of Clinical Pharmacy, Catharina Hospital, Eindhoven, the Netherlands

^2^Department of Clinical Pharmacy and Toxicology, Leiden University Medical Center, Leiden, the Netherlands

^3^Department of Surgical Oncology, Catharina Hospital, Eindhoven, the Netherlands

^4^ GROW, School for Oncology and Development Biology, Maastricht University, Maastricht, the Netherlands

^5^Department of Medical Oncology, Leiden University Medical Center, Leiden, the Netherlands

^6^­Leiden Network for Personalized Therapeutics

*** Correspondence:**M.J. Deenen

maarten.deenen@catharinaziekenhuis.nl

# Search string

**(**(("Oxaliplatin"[majr] OR "oxaliplatin"[ti] OR oxaliplatin*[ti] OR "1,2-Diamminocyclohexane(trans-1)oxolatoplatinum(II)"[ti] OR "Oxaliplatine"[ti] OR "Eloxatine"[ti] OR "Eloxatin"[ti] OR "ACT 078"[ti] OR "ACT-078"[ti] OR "Mitomycin"[majr] OR "mitomycin C"[ti] OR Mitomycin*[ti] OR "ametycine"[ti] OR "mutamycin"[ti] OR "MMC"[ti] OR "hyperthermic intraperitoneal chemotherapy"[ti] OR "hyperthermic intraperitoneal"[ti] OR "hyperthermic intra peritoneal chemotherapy"[ti] OR "hyperthermic intra peritoneal"[ti] OR "HIPEC"[ti]) **AND** ("Genetic Markers"[Mesh] OR "Genetic Marker"[tw] OR "Genetic Markers"[tw] OR "Genetic Biomarker"[tw] OR "Genetic Biomarkers"[tw] OR "DNA Markers"[tw] OR "DNA Marker"[tw] OR "Chromosome Markers"[tw] OR "Chromosome Marker"[tw] OR "Pharmacogenetics"[mesh] OR "pharmacogenetics"[tw] OR pharmacogenetic*[tw] OR "pharmacogenomics"[tw] OR pharmacogenom*[tw] OR "Precision Medicine"[mesh] OR "precision medicine"[tw] OR "individualized"[tw] OR "personalized"[tw] OR "individualised"[tw] OR "personalised"[tw] OR "Polymorphism, Single Nucleotide"[mesh] OR "Single Nucleotide Polymorphism"[tw] OR "Single Nucleotide Polymorphisms"[tw] OR "SNPS"[tw] OR "SNP"[tw] OR "Polymorphism, Genetic"[mesh] OR Polymorphism*[tw] OR "Genetic Markers"[mesh] OR "Genetic Marker"[tw] OR "Genetic Markers"[tw] OR "Genetic Biomarker"[tw] OR "Genetic Biomarkers"[tw] OR "Genes"[mesh] OR "Gene"[tw] OR "Genes"[tw] OR "Mutation"[mesh] OR "Mutation"[tw] OR "Mutations"[tw] OR "DNA Damage"[mesh] OR "DNA Damage"[tw]) **AND** ("Treatment Outcome"[mesh] OR "outcome"[tw] OR "outcomes"[tw])) **OR** (("Oxaliplatin"[majr] OR "oxaliplatin"[ti] OR oxaliplatin*[ti] OR "1,2-Diamminocyclohexane(trans-1)oxolatoplatinum(II)"[ti] OR "Oxaliplatine"[ti] OR "Eloxatine"[ti] OR "Eloxatin"[ti] OR "ACT 078"[ti] OR "ACT-078"[ti] OR "Mitomycin"[majr] OR "mitomycin C"[ti] OR Mitomycin*[ti] OR "ametycine"[ti] OR "mutamycin"[ti] OR "MMC"[ti] OR "hyperthermic intraperitoneal chemotherapy"[ti] OR "hyperthermic intraperitoneal"[ti] OR "hyperthermic intra peritoneal chemotherapy"[ti] OR "hyperthermic intra peritoneal"[ti] OR "HIPEC"[ti]) **AND** ("Genetic Markers"[majr] OR "Genetic Marker"[ti] OR "Genetic Markers"[ti] OR "Genetic Biomarker"[ti] OR "Genetic Biomarkers"[ti] OR "DNA Markers"[ti] OR "DNA Marker"[ti] OR "Chromosome Markers"[ti] OR "Chromosome Marker"[ti] OR "Pharmacogenetics"[majr] OR "pharmacogenetics"[ti] OR pharmacogenetic*[ti] OR "pharmacogenomics"[ti] OR pharmacogenom*[ti] OR "Precision Medicine"[majr] OR "precision medicine"[ti] OR "individualized"[ti] OR "personalized"[ti] OR "individualised"[ti] OR "personalised"[ti] OR "Polymorphism, Single Nucleotide"[majr] OR "Single Nucleotide Polymorphism"[ti] OR "Single Nucleotide Polymorphisms"[ti] OR "SNPS"[ti] OR "SNP"[ti] OR "Polymorphism, Genetic"[majr] OR Polymorphism*[ti] OR "Genetic Markers"[majr] OR "Genetic Marker"[ti] OR "Genetic Markers"[ti] OR "Genetic Biomarker"[ti] OR "Genetic Biomarkers"[ti] OR "Genes"[majr] OR "Gene"[ti] OR "Genes"[ti] OR "Mutation"[majr] OR "Mutation"[ti] OR "Mutations"[ti] OR "DNA Damage"[majr] OR "DNA Damage"[ti]) AND ("Colorectal Neoplasms"[Mesh] OR "colorectal carcinoma"[tw] OR "colorectal carcinomas"[tw] OR "colorectal cancer"[tw] OR "colorectal cancer"[tw] OR "colorectal neoplasm"[tw] OR "colorectal neoplasms"[tw] OR "colorectal tumor"[tw] OR "colorectal tumors"[tw] OR "colorectal tumour"[tw] OR "colorectal tumours"[tw] OR "Adenomatous Polyposis Coli"[tw] OR "Gardner Syndrome"[tw] OR "colorectal carcinoma"[tw] OR "colorectal carcinomas"[tw] OR "colorectal cancer"[tw] OR "colorectal cancer"[tw] OR "colorectal neoplasm"[tw] OR "colorectal neoplasms"[tw] OR "colorectal tumor"[tw] OR "colorectal tumors"[tw] OR "colorectal tumour"[tw] OR "colorectal tumours"[tw] OR "colon carcinoma"[tw] OR "colon carcinomas"[tw] OR "colon cancer"[tw] OR "colon cancer"[tw] OR "colon neoplasm"[tw] OR "colon neoplasms"[tw] OR "colon tumor"[tw] OR "colon tumors"[tw] OR "colon tumour"[tw] OR "colon tumours"[tw] OR "colonic carcinoma"[tw] OR "colonic carcinomas"[tw] OR "colonic cancer"[tw] OR "colonic cancer"[tw] OR "colonic neoplasm"[tw] OR "colonic neoplasms"[tw] OR "colonic tumor"[tw] OR "colonic tumors"[tw] OR "colonic tumour"[tw] OR "colonic tumours"[tw] OR "sigmoid carcinoma"[tw] OR "sigmoid carcinomas"[tw] OR "sigmoid cancer"[tw] OR "sigmoid cancer"[tw] OR "sigmoid neoplasm"[tw] OR "sigmoid neoplasms"[tw] OR "sigmoid tumor"[tw] OR "sigmoid tumors"[tw] OR "sigmoid tumour"[tw] OR "sigmoid tumours"[tw] OR "rectal carcinoma"[tw] OR "rectal carcinomas"[tw] OR "rectal cancer"[tw] OR "rectal cancer"[tw] OR "rectal neoplasm"[tw] OR "rectal neoplasms"[tw] OR "rectal tumor"[tw] OR "rectal tumors"[tw] OR "rectal tumour"[tw] OR "rectal tumours"[tw] OR "rectum carcinoma"[tw] OR "rectum carcinomas"[tw] OR "rectum cancer"[tw] OR "rectum cancer"[tw] OR "rectum neoplasm"[tw] OR "rectum neoplasms"[tw] OR "rectum tumor"[tw] OR "rectum tumors"[tw] OR "rectum tumour"[tw] OR "rectum tumours"[tw] OR "anus carcinoma"[tw] OR "anus carcinomas"[tw] OR "anus cancer"[tw] OR "anus cancer"[tw] OR "anus neoplasm"[tw] OR "anus neoplasms"[tw] OR "anus tumor"[tw] OR "anus tumors"[tw] OR "anus tumour"[tw] OR "anus tumours"[tw] OR "anal carcinoma"[tw] OR "anal carcinomas"[tw] OR "anal cancer"[tw] OR "anal cancer"[tw] OR "anal neoplasm"[tw] OR "anal neoplasms"[tw] OR "anal tumor"[tw] OR "anal tumors"[tw] OR "anal tumour"[tw] OR "anal tumours"[tw] OR "anal gland carcinoma"[tw] OR "anal gland carcinomas"[tw] OR "anal gland cancer"[tw] OR "anal gland cancer"[tw] OR "anal gland neoplasm"[tw] OR "anal gland neoplasms"[tw] OR "anal gland tumor"[tw] OR "anal gland tumors"[tw] OR "anal gland tumour"[tw] OR "anal gland tumours"[tw]))**)**

# Tables

Table S1. Overview of studies on the association between *ERCC1* biomarkers and treatment outcome of oxaliplatin-based chemotherapy in CRC patients

|  |  |  |  |  |  |  |  |  | *Univariate analysis* | | | *Multivariate analysis* | | |
| --- | --- | --- | --- | --- | --- | --- | --- | --- | --- | --- | --- | --- | --- | --- |
| *Author,*  *year* | *n* | *CRC type* | *Treatment* | *Biomarker* | *rs number* | *Type of sample* | *Type of assay* | *Reference*/comparator (n)* | *PFS* | *DFS* | *OS* | *PFS* | *DFS* | *OS* |
| Rao et al., 2019 [26] | 24 | stage II-III mCRC | CAPOX or FOLFOX | ERCC1-118 | n.a. | blood | protein expression | **underexpression**  **+normal (11)**  overexpression (13) | HR=2.35  (95% CI:  1.00-5.48)  p=0.02 |  |  |  |  |  |
| Kassem et al.,  2017 [44] | 65 | stage III-IV | CAPOX or FOLFOX-4 | *ERCC1* | n.a. | tumor tissue | mRNA expression | **low (50)**  high (15) |  |  | HR=2.80  (95% CI:  1.27–6.21)  p=0.01 |  |  |  |
| Basso et al.,  2013 [45] | 60 | mCRC | FOLFOX-6 | *ERCC1* | n.a. | normal and tumor tissue | mRNA expression | **overexpression (30)**  underexpression (30) | HR=1.09  (95% CI:  0.63–1.95)  p=0.71 |  |  |  |  |  |
| Sfakianaki et al.,  2019 [46] | 246 | stage II-III | CAPOX or FOLFOX | *ERCC1* | n.a. | tumor tissue | mRNA expression | **low (118)**  high (128) |  | HR=1.16  (95% CI:  0.61-1.57)  p=0.93 | HR=1.00  (95% CI:  0.56-1.80)  p=0.99 |  |  |  |
| Li et al.,  2014 [47] | 112 | stage II-III | FOLFOX-4 or mFOLFOX or CAPOX | *ERCC1* | n.a. | tumor tissue | mRNA expression | **low (-)**  high (-) |  | HR=1.05  (95% CI:  0.85-1.30)  p=0.64 |  |  |  |  |
| Monzo et al.,  2007 [43] | 42 | aCRC | CAPOX | ERCC1-Lys259Thr  *c.776A>C* | rs735482 | blood | polymorphism | A/A (33)  A/C (4) + C/C (5) |  |  | 14.4 mo vs  30.0 mo  p=0.55 |  |  |  |
| Huang et al.,  2011 [25] | 157 | mCRC | FOLFOX-4 | ERCC1-Asn118=  *c.354T>C* | rs11615 | blood | polymorphism | **T/T (19)**  C/T (58  C/C (80) | C/C  HR=0.06  (95% CI:  0.01–0.27)  p<0.01 C/T  HR=0.48  (95% CI:  0.13–1.74)  p=0.26 |  | C/C  HR=0.07  (95% CI:  0.01–0.38)  p<0.01 C/T  HR=0.39  (95% CI:  0.08–1.89)  p=0.25 |  |  |  |
| Rao et al.,  2019 [26] | 97 | stage II-III mCRC | CAPOX or FOLFOX | ERCC1-Asn118=  *c.354T>C* | rs11615 | tumor tissue | polymorphism | C/C (42)  T/C (40)  T/T (15) | 211 days vs  196 days vs  590 days  p=0.03 |  |  |  |  |  |
| Li et al.,  2012 [27] | 335 | aCRC | FOLFOX-6 | ERCC1-Asn118=  *c.354T>C* | rs11615 | blood | polymorphism | **T/T (166)**  T/C (140)  C/C (29) |  |  | T/C  HR=0.87  (95% CI:  0.60-1.26) C/C  HR= 0.22  (95% CI:  0.12-0.81) |  |  | T/C  HR=0.81  (95% CI:  0.52-1.14)  p=0.16 C/C  HR=0.20  (95% CI:  0.10-0.79)  p<0.05 |
| Lamas et al.,  2011 [28] | 72 | aCRC | mFOLFOX-6 | ERCC1-Asn118=  *c.354T>C* | rs11615 | blood | polymorphism | C/C  C/T  T/T | 9 mo  vs  10 mo  vs  10 mo  p=1.0 |  |  |  |  |  |
| van Huis-Tanja et al.,  2014 [29] | 145 | aCRC | CAPOX | ERCC1-Asn118=  *c.354T>C* | rs11615 | blood | polymorphism | T/T (59)  C/T (72)  C/C (14) | 4.2 mo  vs  4.2 mo  vs  4.5 mo  p=0.19 |  | 10.0 mo vs  12.1 mo vs  10.8 mo  p=0.19 |  |  |  |
| Zaanan et al.,  2014 [30] | 202 | stage III | FOLFOX-4 or FOLFOX-6 | ERCC1-Asn118=  *c.354T>C* | rs11615 | tumor tissue | polymorphism | **C/C (49)**  C/T (88) + T/T (65) |  | HR=2.29  (95% CI:  0.97-5.41)  p= 0.06 |  |  |  |  |
| Stoehlmacher et al.,  2004 [31] | 106 | mCRC | FUOX | ERCC1-Asn118=  *c.354T>C* | rs11615 | blood | polymorphism | **C/C (30)**  C/T (45)  T/T (31) | C/T  RR=1.24  (95% CI:  0.73- 2.11)  T/T  RR=1.36  (95% CI:  0.76-2.41)  p=0.51 |  | C/T  RR=2.29  (95% CI:  1.19- 4.41)  T/T  RR=1.86  (95% CI:  0.91-3.83)  p=0.02 |  |  | C/T + T/T  RR=2.05  (95% CI:  1.00-4.20)  p=0.04 |
| Liang et al.,  2010 [32] | 113 | mCRC | mFOLFOX-4 or CAPOX | ERCC1-Asn118=  *c.354T>C* | rs11615 | blood | polymorphism | **C/C (55)**  C/T (43)  T/T (15) |  |  |  |  |  | C/T  HR=1.46  (95% CI:  0.94-2.27)  p=0.10 T/T  HR=1.66  (95% CI:  0.91, 3.01)  p=0.10 |
| Paré et al.,  2008 [33] | 106 | mCRC | FOLFOX | ERCC1-Asn118=  *c.354T>C* | rs11615 | leukocytes | polymorphism | **T/T (42) + C/T (52)**  C/C (24) | 10 mo vs  6 mo  p<0.001 |  | 30 mo vs  11 mo  p<0.01 |  |  | RR=1.8  (CI 95%:  1.1–3.0)  p=0.02 |
| Martinez-Balibrea et al., 2008 [34] | 47 | mCRC | XELOX | ERCC1-Asn118=  *c.354T>C* | rs11615 | blood | polymorphism | **T/T (18)**  C/T + C/C (29) | HR=1.13  (95% CI:  0.57–2.24)  p=0.74 |  |  |  |  |  |
| Martinez-Balibrea et al., 2008 [34] | 49 | mCRC | FUOX | ERCC1-Asn118=  *c.354T>C* | rs11615 | blood | polymorphism | **T/T (21)**  T/C + C/C (28) | HR=1.96  (95% CI:  0.99–3.92)  p=0.05 |  |  | HR=2.12 (95% CI: 1.05–4.28)  p=0.04 |  |  |
| Chang et al.,  2009 [35] | 168 | mCRC | FOLFOX-4 | ERCC1-Asn118=  *c.354T>C* | rs11615 | blood | polymorphism | **T/T (21) + C/T (67)**  C/C (80) | 7 vs  13 mo  p<0.01 |  | 16 mo vs  25 mo  p<0.01 |  |  |  |
| Chen et al.,  2010 [36] | 166 | mCRC | FOLFOX-4 | ERCC1-Asn118=  *c.354T>C* | rs11615 | blood | polymorphism | **C/C (78)**  C /T + T/T (88) |  |  |  |  |  | HR=3.15  (95% CI:  1.89–5.23)  p<0.01 |
| Nishina et al., 2013 [38] | 68 | aCRC and/or recurrent CRC | mFOLFOX-6 + bevacizumab | ERCC1-Asn118=  *c.354T>C* | rs11615 | blood | polymorphism | **C/C (29)**  C/T + T/T (39) | 13.5 mo vs  12.6 mo  HR=1.08 p=0.80 |  |  |  |  |  |
| Chua et al.,  2009 [39] | 115 | mCRC | FOLFOX | ERCC1-Asn118=  *c.354T>C* | rs11615 | tumor tissue | polymorphism | **C/C (10)** C/T (64)  T/T (41) | C/T  HR=2.68 (95% CI: 1.15-6.23) p=0.02 T/T  HR=2.54 (95% CI: 1.07-6.04) p=0.04 C/T + T/T HR=2.62 (95% CI: 1.14-6.02) p=0.02 |  | C/T HR=1.88 (95% CI: 0.75-4.71) p=0.20 T/T HR=1.55 (95% CI: 0.60-4.00) p=0.40 C/T + T/T HR=1.74 (95% CI: 0.70-4.30) p=0.20 | C/T + T/T HR=2.16 (95% CI: 0.94-4.97) p=0.07 |  |  |
| Ruzzo et al.,  2007 [40] | 166 | mCRC | FOLFOX-4 | ERCC1-Asn118=  *c.354T>C* | rs11615 | blood | polymorphism | **C/C (31)**  C/T (85)  T/T (50) | C/T HR=1.23  (95% CI:  0.78-1.94)  p=0.27 T/T  HR=0.53  (95% CI:  1.51-4.25)  p<0.01 |  |  | C/T  HR=1.32  (95% CI:  0.78-2.24)  p=0.29 T/T  HR=2.34  (95% CI:  1.28-4.27)  p<0.01 |  |  |
| Sarasqueta et al., 2011 [41] | 48 | stage III | CAPOX or FOLFOX | ERCC1-Asn118=  *c.354T>C* | rs11615 | normal tissue | polymorphism | **T/T -**  T/C -  C/C - |  |  |  |  | T/C  HR=0.67  (95% CI:  0.23-1.89)  p=0.45 C/C  HR=0.94  (95% CI:  0.26-3.36)  p=0.92 |  |
| Kumamoto et al., 2013 [42] | 63 | n.s. | mFOLFOX-6 | ERCC1-Asn118=  *c.354T>C* | rs11615 | blood | polymorphism | **C/C (30)**  C/T (23) T/T (10) | 9.9 mo vs  8.1 mo vs  8.3 mo  p=0.63 |  | 27.4 mo vs  22.5 mo vs  32.9 mo  p=0.38 |  |  |  |
| Nishina et al., 2013 [38] | 68 | aCRC and/or recurrent CRC | mFOLFOX-6 + bevacizumab | ERCC1-Gln504Lys  c.1516C>A | rs3212986 | blood | polymorphism | **C/C (41)**  C/A + A/A (27) | 13.8 mo vs  12.6 mo  HR=1.18  p=0.71 |  |  |  |  |  |
| Huang et al.,  2011 [25] | 157 | mCRC | FOLFOX-4 | ERCC1-Asn118=  *c.354T>C* and XRCC1- Gln399Arg  *c.1196A>G* | rs11615 and rs25487 | blood | polymorphism | 2 favorable genotypes (*ERCC1* C/C and *XRCC1* G/G) - vs  ≤1 favorable genotype - |  |  | 25 mo vs  16.5 mo  p<0.01 |  |  |  |
| Liang et al.,  2010 [32] | 113 | mCRC | Modified FOLFOX-4 or CAPOX | ERCC1-Asn118=  *c.354T>C* and XRCC1- Gln399Arg  *c.1196A>G* | rs11615 and rs25487 | blood | polymorphism | **2 favorable genotypes (*XRCC1* A/A and *ERCC1* C/C) 38** vs  1 favorable genotype 40 vs  0 favorable genotype 35 |  |  |  |  |  | 1  HR=2.25  (95% CI:  1.38-3.67)  p=0.01  0  HR=2.60  (95% CI:  1.56-4.31)  p<0.01 |
| Zaanan et al.,  2014 [30] | 210 | stage III | FOLFOX-4 or FOLFOX-6 | ERCC1-Asn118=  *c.354T>C* and XRCC1- Gln399Arg  *c.1196A>G* | rs11615 and rs25487 | tumor tissue | polymorphism | **≥ 1 favorable genotype (*ERCC1* C/C and/or *XRCC1* G/G + G/A)** vs  0 favorable genotype |  | HR=2.42 (95% CI: 1.16-5.03) p=0.02 |  |  | HR=2.03  (95% CI:  0.96-4.28) p=0.06 |  |

Abbreviations: aCRC, advanced colorectal cancer; CAPOX, capecitabine and oxaliplatin; CI, confidence interval; *ERCC1, excision repair cross-complementing group 1;* DFS, disease-free survival; FOLFOX, 5-fluorouracil, leucovorin and oxaliplatin; FUOX, 5-fluorouracil and oxaliplatin; HR, hazard ratio; mo, months; n.a., not applicable; n.s., not specified; OR, odds ratio; OS, overall survival; PFS, progression-free survival; RR, relative risk; *XRCC1, X-ray repair cross-complementation group 1*. *Reference group in bold.

Table S2. Overview of studies on the association between *XPA* biomarkers and treatment outcome of oxaliplatin-based chemotherapy in CRC patients

|  |  |  |  |  |  |  |  |  | *Univariate analysis* | | | *Multivariate analysis* | |
| --- | --- | --- | --- | --- | --- | --- | --- | --- | --- | --- | --- | --- | --- |
| *Author,*  *year* | ***n*** | ***CRC type*** | ***Treatment*** | ***Biomarker*** | ***rs number*** | ***Type of sample*** | ***Type of assay*** | ***Reference*/comparator (n)*** | ***PFS*** | ***DFS*** | ***OS*** | ***DFS*** | ***OS*** |
| Hu et al., 2019 [51] | 580 | aCRC | FOLFOX4 or CAPOX | XPA  *g.100452435C>T* | rs2808668 | blood | polymorphism | **C/C –**  TT + C/T - |  |  |  | HR=1.19  (95% CI:  0.90–1.57)  p=0.22 | HR=1.17 (95% CI: 0.88–1.54)  p=0.28 |
| Hu et al., 2019 [51] | 580 | stage III - IV | FOLFOX4 or CAPOX | XPA  *g.100462409T>C* | rs10817938 | blood | polymorphism | **T/T (306)**  C/C + C/T (274) |  | 46 mo vs  48 mo  p=0.06 | 55 mo vs  62 mo p<0.01 | HR=0.79  (95% CI:  0.63–1.00)  p=0.05 | HR=0.73 (95% CI: 0.58–0.92) p=0.01 |
| Stoehlmacher et al.,  2004 [31] | 93 | rCRC | FUOX | *XPA*  *c.-4A>G* | rs1800975 | blood | polymorphism | **G/G (24)**  A/G (53)  A/A (16) | A/G  RR=1.24  (95% CI:  0.70-2.18)  A/A  RR=1.37  (95% CI:  0.66-2.84) p=0.61 |  | A/G RR=0.88 (95% CI: 0.47-1.62)  A/A RR=1.13 (95% CI: 0.49-2.45) p=0.76 |  |  |
| Monzo et al., 2007 [43] | 42 | aCRC | CAPOX | *XPA*  *c.-4A>G* | rs1800975 | blood | polymorphism | A/A (17)  G/A (20) + A/A (5) |  |  | 19.2 mo vs 18.1 mo  p=0.29 |  |  |
| Monzo et al., 2007 [43] | 42 | aCRC | CAPOX | XPG-His46=  *c.138T>C*  and  *XPA*  *c.-4A>G* | rs1047768 + rs1800975 | blood | polymorphism | **Favorable genotype (*XPG* (C/C) + *XPA* (G/A or G/G))** vs  unfavorable genotype |  |  | 49.6 mo vs  14.0 mo p<0.01 |  | RR=34  (95% CI: 6.3–183)  p<0.01 |

Abbreviations: aCRC, advanced colorectal cancer; CAPOX, capecitabine and oxaliplatin; CI, confidence interval; DFS, disease-free survival; FOLFOX, 5-fluorouracil, leucovorin and oxaliplatin; FUOX, 5-fluorouracil and oxaliplatin; HR, hazard ratio; mo, months; OS, overall survival; PFS, progression-free survival; rCRC, refractory colorectal cancer; RR, relative risk; *XPA, xeroderma pigmentosum complementation group A*; *XPG, xeroderma pigmentosum complementation group G*. *Reference group in bold.

Table S3. Overview of studies on the association between *XPC* biomarkers and treatment outcome of oxaliplatin-based chemotherapy in CRC patients

|  |  |  |  |  |  |  |  |  |  | *Univariate analysis* | *Multivariate analysis* | |
| --- | --- | --- | --- | --- | --- | --- | --- | --- | --- | --- | --- | --- |
| *Author,*  *year* | ***n*** | ***CRC type*** | ***Treatment*** | ***Biomarker*** | ***rs number*** | ***Type of sample*** | ***Type of assay*** |  | ***Reference*/comparator (n)*** | ***OS*** | ***DFS*** | ***OS*** |
| Liu et al., 2012 [49] | 432 | n.s. | CAPOX or FOLFOX | XPC-Gln939Lys  *c.2815C>A* | rs2228001 | blood | polymorphism |  | **A/A -**  A/C + C/C - | HR=0.97  (95% CI:  0.75–1.32)  p=0.99 |  |  |
| Kap et al., 2015 [50] | 201 | stage II - IV | Oxaliplatin-based chemotherapy | *XPC*  *c.*463A>G* | rs1043953 | blood/  saliva | polymorphism |  | **AA -**  AG + GG - | HR=0.45  (95%CI:  0.29–0.70)  p<0.01 |  |  |
| Hu et al., 2019 [51] | 580 | stage III - IV | CAPOX or FOLFOX4 | *XPC*  *c.-27G>C* | rs2607775 | blood | polymorphism |  | **C/C -**  C/G + G/G |  | HR=0.91  (95% CI:  0.70–1.17)  p=0.44 | HR=0.91  (95% CI:  0.70–1.17)  p=0.46 |

Abbreviations: CAPOX, capecitabine and oxaliplatin; CI, confidence interval; CRC, colorectal cancer; FOLFOX, 5-fluorouracil, leucovorin and oxaliplatin; HR, hazard ratio; n.s., not specified; OS, overall survival; PFS, progression-free survival; *XPC, xeroderma pigmentosum complementation group C*. *Reference group in bold.

Table S4. Overview of studies on the association between *XPD* biomarkers and treatment outcome of oxaliplatin-based chemotherapy in CRC patients

|  |  |  |  |  |  |  |  |  | *Univariate analysis* | | *Multivariate analysis* | | |
| --- | --- | --- | --- | --- | --- | --- | --- | --- | --- | --- | --- | --- | --- |
| *Author,*  *year* | *n* | *CRC type* | *Treatment* | *Biomarker* | *rs number* | *Type of sample* | *Type of assay* | *Reference*/comparator (n)* | *PFS* | *OS* | *PFS* | *DFS* | *OS* |
| Kassem et al.,  2017 [44] | 64 | stage III- IV | CAPOX or FOLFOX | *XPD* | n.a. | tumor tissue | mRNA expression | **low (48)**  high (16) |  | HR=1.36  (95% CI:  0.59-3.14)  p= 0.47 |  |  |  |
| Kjersem et al.,  2015 [54] | 508 | mCRC | FOLFOX or Nordic FLOX + cetuximab | XPD-Arg156=  *c.468A>C* | rs238406 | blood | polymorphism | C/C (173) + C/A (233)  A/A (102) | 7.8 mo vs  9.1 mo  p<0.01 | 23.4 mo vs  20.3 mo  p=0.33 |  |  |  |
| Stoehlmacher et al.,  2004 [31] | 103 | rCRC | FUOX | XPD-Arg156=  *c.468A>C* | rs238406 | blood | polymorphism | **A/A (14)**  C/A (59)  C/C (30) | C/A  RR=0.81  (95% CI:  0.44-1.49)  C/C  RR=0.73  (95% CI:  0.36-1.48)  p=0.64 | C/A  RR=1.22  (95% CI:  0.55-2.75)  C/C  RR=1.18  (95% CI:  0.49-2.82)  p=0.88 |  |  |  |
| Park et al., 2001 [55] | 69 | rCRC | FUOX | XPD-Arg156=  *c.468A>C* | rs238406 | blood | polymorphism | **C/C (22)**  C/A (38)  A/A (9) |  | 11.7 mo vs  13.2 mo vs  8.5 mo  C/A  RR=0.94 A/A  RR=1.60  p=0.50 |  |  |  |
| Liu et al., 2019 [56] | 106 | stage IV | mFOLFOX4 or CAPOX | XPD-Asp312Asn  *c.934G>A* | rs1799793 | blood | polymorphism | **G/G (49)**  G/A (42)  A/A (15) |  | G/A  HR=1.26  (95% CI:  0.83–1.91)  p=0.28 A/A  HR=1.65  (95% CI:  0.92–2.97)  p=0.09 G/A + A/A  HR=1.34  (95% CI:  0.91–1.98)  p=0.14 |  |  | G/A  HR=1.51  (95% CI:  0.98–2.34)  p=0.06 A/A  HR=2.43  (95% CI:  1.31–4.53)  p<0.01 |
| Park et al., 2001 [55] | 59 | rCRC | FUOX | XPD-Asp312Asn  *c.934G>A* | rs1799793 | blood | polymorphism | **A/A (7)**  G/A (24)  G/G (28) |  | Not reached  9.2 mo vs  26.5 mo  G/A  RR=2.17  G/G  RR=1.29  p=0.27 |  |  |  |
| Ruzzo et al., 2007 [40] | 165 | mCRC | FOLFOX-4 | XPD-Asp312Asn  *c.934G>A* | rs1799793 | blood | polymorphism | **G/G (57)**  G/A (86)  A/A (22) | G/A  HR=1.02  (95% CI:  0.62-1.69)  p=0.93 A/A  HR=1.40  (95% CI:  0.83-2.37)  p=0.21 |  | G/A  HR=1.13  (95% CI:  0.73-1.78)  p=0.58 A/A  HR=1.65  (95% CI:  0.73-3.21)  p=0.12 |  |  |
| Sarasqueta et al.,  2011 [41] | 43 | stage III | CAPOX or FOLFOX | XPD-Lys751Gln  *c.2251A>C* | rs13181 | normal tissue | polymorphism | **A/A -**  A/C -  C/C - |  |  |  | AC  HR=0.65  (95% CI:  0.24-1.8)  p=0.41 CC HR=0.73  (95% CI:  0.13-4.11)  p=0.72 |  |
| Lamas et al., 2011 [28] | 72 | aCRC | mFOLFOX-6 | XPD-Lys751Gln  *c.2251A>C* | rs13181 | blood | polymorphism | A/A 28  A/C 33  C/C 11 | 8 mo vs  16 mo vs  10 mo  p=0.02 |  |  |  |  |
| Gan et al., 2012 [59] | 289 | aCRC | FOLFOX | XPD-Lys751Gln  *c.2251A>C* | rs13181 | blood | polymorphism | **A/A (138)**  A/C (125)  C/C (26) |  | A/C  HR=0.91  (95% CI:  0.66-1.87) C/C  HR=0.51  (95% CI:  0.33 -0.94) |  |  |  |
| Li et al.,  2012 [27] | 335 | aCRC | FOLFOX-6 | XPD-Lys751Gln  *c.2251A>C* | rs13181 | blood | polymorphism | **A/A (153)**  A/C (150)  C/C (32) |  | A/C  HR=0.88  (95% CI:  0.61-1.28) C/C  HR=0.52  (95% CI:  0.23-1.09) |  |  | A/C  HR=0.86  (95% CI:  0.56-1.20)  p=0.48  C/C  HR=0.48  (95% CI:  0.19-0.97)  p<0.05 |
| Huang et al., 2011 [25] | 157 | mCRC | FOLFOX-4 | XPD-Lys751Gln  *c.2251A>C* | rs13181 | blood | polymorphism | **C/C (1)**  A/C (19)  A/A (137) | A/C  HR=0.76  (95% CI:  0.09–6.58)  p=0.81  A/A  HR=0.28  (95% CI:  0.03–2.38)  p=0.24 | A/C  HR=0.86  (95% CI:  0.10–7.74)  p=0.86  A/A  HR=0.30  (95% CI:  0.03–2.82)  p=0.30 |  |  |  |
| Le Morvan et al.,  2007 [57] | 59 | mCRC | oxaliplatin-based chemotherapy | XPD-Lys751Gln  *c.2251A>C* | rs13181 | blood | polymorphism | A/C (33) + C/C (6)  A/A (20) |  | 15.6 mo vs  26.3 mo  p=0.02 |  |  |  |
| Stoehlmacher et al.,  2004 [31] | 106 | rCRC | FUOX | XPD-Lys751Gln  *c.2251A>C* | rs13181 | blood | polymorphism | **A/A (40)**  A/C (53)  C/C (13) | A/C  RR=1.13 (95% CI: 0.72-1.78) C/C  RR=1.25 (95% CI: 0.59-2.67)  p=0.76 | A/C  RR=1.87  (95% CI:  1.06-3.31)  C/C  RR=2.44  (95% CI:  1.09-5.44)  p=0.05 |  |  | A/C  RR=1.50  (95% CI:  0.79- 2.87)  C/C  RR=3.33  (95% CI:  1.39-7.99)  p=0.04 |
| Paré et al., 2008 [33] | 121 | mCRC | FOLFOX | XPD-Lys751Gln  *c.2251A>C* | rs13181 | leukocytes | polymorphism | **A/A (52)**  A/C (45) + C/C (24) | 12 mo vs  8 mo  p<0.01 | 41 mo vs  17 mo  p=0.02 | RR=1.7  (95% CI: 1.1–2.8) p=0.02 |  | RR=1.6  (95% CI:  1.1–2.5)  p=0.03 |
| Martinez-Balibrea et al.,  2008 [34] | 47 | mCRC | CAPOX | XPD-Lys751Gln  *c.2251A>C* | rs13181 | blood | polymorphism | **A/A (20)**  A/C (21)  C/C (6) | A/C  HR=1.05  (95% CI:  0.51–2.15)  C/C  HR=0.54  (95% CI:  0.19–1.52)  p=0.39 |  |  |  |  |
| Martinez-Balibrea et al.,  2008 [34] | 48 | mCRC | FUOX | XPD-Lys751Gln  *c.2251A>C* | rs13181 | blood | polymorphism | **A/A (22)**  A/C (19)  C/C (8) | A/C  HR=0.94  (95% CI:  0.45–1.97)  C/C  HR=1.50  (95% CI:  0.61–3.69)  p=0.61 |  |  |  |  |
| Chen et al., 2010 [36] | 166 | mCRC | FOLFOX-4 | XPD-Lys751Gln  *c.2251A>C* | rs13181 | blood | polymorphism | **A/A (139)**  A/C (27) |  |  |  |  | HR=4.41  (95% CI:  2.51–7.75)  p<0.01 |
| Etienne‐Grimaldi et al.,  2010 [60] | 115 | aCRC | mFOLFOX7 | XPD-Lys751Gln  *c.2251A>C* | rs13181 | blood | polymorphism | A/A (41)  A/C (58)  C/C (16) | 6.4 mo vs  8.0 mo vs  6.4 mo  p=0.33 |  |  |  |  |
| Lai et al., 2009 [58] | 188 | mCRC | FOLFOX-4 | XPD-Lys751Gln  *c.2251A>C* | rs13181 | blood | polymorphism | A/A (158)  A/C (30) | 11 mo vs  7 mo  p<0.01 | 22 mo vs  14 mo  p<0.01 |  |  |  |
| Park et al., 2001 [55] | 71 | mCRC | FUOX | XPD-Lys751Gln  *c.2251A>C* | rs13181 | blood | polymorphism | **A/A (22)**  A/C (39)  C/C (10) |  | 17.4 mo vs  12.8 mo vs  3.3 mo  A/C  RR=1.31 C/C  RR=4.01  p<0.01 |  |  |  |
| Monzo et al., 2007 [43] | 42 | aCRC | CAPOX | XPD-Lys751Gln  *c.2251A>C* | rs13181 | blood | polymorphism | A/A (21)  A/C (17) + C/C (4) | 14.4 mo vs  19.2 mo  p=0.83 | 8.3 mo vs 8.4 mo, p=0.54 |  |  |  |
| Ruzzo et al., 2007 [40] | 165 | mCRC | FOLFOX-4 | XPD-Lys751Gln  *c.2251A>C* | rs13181 | blood | polymorphism | **A/A (43)**  A/C (97)  C/C (25) | A/C  HR=1.67  (95% CI:  0.96-2.89)  p=0.06 C/C  HR=1.79  (95% CI:  1.13-3.09)  p=0.03 |  | A/C  HR=1.81  (95% CI:  1.01-3.25)  p=0.04 C/C  HR=2.21  (95% CI:  1.17-4.17)  p=0.01 |  |  |
| Kumamoto et al.,  2013 [42] | 63 | n.s. | mFOLFOX-6 | XPD-Lys751Gln  *c.2251A>C* | rs13181 | blood | polymorphism | **A/A (58)**  A/C (5) | 10.3 mo vs  6.1 mo  p=0.05 | 25.5 mo vs  29.2mo  p=0.26 |  |  |  |

Abbreviations: aCRC, advanced colorectal cancer; CAPOX, capecitabine and oxaliplatin; CI, confidence interval; DFS, disease-free survival; FOLFOX, 5-fluorouracil, leucovorin and oxaliplatin; FLOX, 5-fluorouracil and folinic acid; FUOX, 5-fluorouracil and oxaliplatin; HR, hazard ratio; mCRC, metastatic colorectal cancer; mo, months; n.s., not specified; OR, odds ratio OS, overall survival; PFS, progression-free survival; rCRC, refractory colorectal cancer; RR, relative risk; *XPG, xeroderma pigmentosum complementation group G*. *Reference group in bold.

Table S5. Overview of studies on the association between *XPG* biomarkers and treatment outcome of oxaliplatin-based chemotherapy in CRC patients

|  |  |  |  |  |  |  |  |  | *Univariate analysis* | | *Multivariate analysis* | |
| --- | --- | --- | --- | --- | --- | --- | --- | --- | --- | --- | --- | --- |
| *Author,*  *year* | *n* | *CRC type* | *Treatment* | *Biomarker* | *rs number* | *Type of sample* | *Type of assay* | *Reference*/comparator (n)* | *PFS* | *OS* | *PFS* | *OS* |
| Monzo et al.,  2007 [43] | 42 | aCRC | CAPOX | XPG-His46=  *c.138T>C* | rs1047768 | blood | polymorphism | C/C (19)  C/T (19) + TT (4) |  | 32.2 mo vs  12.0 mo  p<0.01 |  |  |
| Kweekel et al.,  2009 [65] | 91 | aCRC | CAPOX | XPG-His46=  *c.138T>C* | rs1047768 | blood | polymorphism | **T/T (28)**  T/C (46)  C/C (17) |  | No difference | T/C  HR=1.71  (95% CI:  0.98-2.98) C/C  HR=2.85  (95% CI:  1.42-5.71)  p<0.01 |  |
| Chen et al., 2016 [64] | 170 | aCRC | FOLFOX | *XPG*  *+25A>G* | n.s. | blood | polymorphism | **AA (32) + AG (83)**  GG (55) | HR=1.59  (95% CI:  1.14–2.22)  p<0.01 | HR= 1.58  (95%CI:  1.14–2.22)  p<0.01 | HR=1.50  (95% CI:  1.07–2.11)  p=0.02 | HR=1.68  (95% CI:  1.18–2.39)  p<0.01 |
| Liu et al., 2012 [49] | 432 | n.s. | CAPOX or FOLFOX | XPG- Asp1104His  *c.3310G>C* | rs17655 | blood | polymorphism | **G/G**  G/C + C/C |  | HR=1.43  (95%CI:  1.01–2.00)  p=0.04 |  | HR 1.69  (95%CI:  1.20–2.38)  p<0.01 |
| Chen et al., 2016 [64] | 170 | aCRC | FOLFOX | *XPG*  *-763A>G* | n.s. | blood | polymorphism | **GG (35) + GA (78)**  AA (57) | HR=1.75  (95% CI:  1.14–2.22)  p<0.01 | HR=1.73  (95%CI:  1.24–2.40)  p<0.01 | HR=1.72  (95% CI:  1.23–2.41)  p<0.01 | HR=1.88  (95% CI:  1.33–2.66)  p<0.01 |
| Monzo et al.,  2007 [43] | 42 | aCRC | CAPOX | XPG-His46=  *c.138T>C*  and  *XPA*  c*.-4A>G* | rs1047768 and rs1800975 | blood | polymorphism | **Favorable genotype (*XPG* (C/C) + *XPA* (G/A or G/G)** vs  unfavorable genotype |  | 49.6 mo vs  7.8 mo  p<0.01 |  | RR=34  (95%  CI:  6.3-183)  p<0.01 |

Abbreviations: aCRC, advanced colorectal cancer; CAPOX, capecitabine and oxaliplatin; CI, confidence interval; CRC, colorectal cancer, DFS, disease-free survival; FOLFOX, 5-fluorouracil; leucovorin and oxaliplatin; HR, hazard ratio; mo, months; n.s., not specified; OS, overall survival; OR, odds ratio; PFS, progression-free survival; *XPA, xeroderma pigmentosum complementation group A*; *XPG, xeroderma pigmentosum complementation group G*. *Reference group in bold.

Table S6. Overview of studies on the association between *MNAT1* biomarkers and treatment outcome of oxaliplatin-based chemotherapy in CRC patients

| *Author,*  *year* | *n* | *CRC type* | *Treatment* | *Biomarker* | *rs number* | *Type of sample* | *Type of assay* | *Reference*/comparator (n)* | *Univariate analysis*  *OS* |
| --- | --- | --- | --- | --- | --- | --- | --- | --- | --- |
| Kap et al., 2015 [50] | 192 | stage II - IV | oxaliplatin-based chemotherapy | *MNAT1*  *c.688-30168A>G* | rs3783819 | blood/  saliva | polymorphism | **A/A -** vs  G/A + G/G - | HR=0.51  (95% CI:  0.36–0.73)  p<0.01 |
| Kap et al., 2015 [50] | 201 | stage II - IV | oxaliplatin-based chemotherapy | *MNAT1*  *c.562-88A>G* | rs973063 | blood/  saliva | polymorphism | **A/A -** vs  G/A + G/G - | HR=0.52  (95% CI:  0.37–0.72)  p<0.01 |
| Kap et al., 2015 [50] | 201 | stage II - IV | oxaliplatin-based chemotherapy | *MNAT1*  *c.809+24992A>G* | rs4151330 | blood/  saliva | polymorphism | **A/A -** vs  G/A + G/G - | HR=0.53  (95% CI:  0.38–0.75)  p<0.01 |

Abbreviations: CI, confidence interval; CRC, colorectal cancer; HR, hazard ratio; OS, overall survival. *Reference group in bold.

Table S7. Overview of studies on the association between *XRCC1* biomarkers and treatment outcome of oxaliplatin-based chemotherapy in CRC patients

|  |  |  |  |  |  |  |  |  | *Univariate analysis* | | | *Multivariate analysis* | |
| --- | --- | --- | --- | --- | --- | --- | --- | --- | --- | --- | --- | --- | --- |
| *Author,*  *year* | *n* | *CRC type* | *Treatment* | *Biomarker* | *rs number* | *Type of sample* | *Type of assay* | *Reference*/ comparator (n)* | *PFS* | *DFS* | *OS* | *DFS* | *OS* |
| Huang et al., 2011 [25] | 157 | mCRC | FOLFOX-4 | XRCC1- Gln399Arg  *c.1196A>G* | rs25487 | blood | polymorphism | **A/A (10)**  G/A (57)  G/G (90) | G/G  HR=0.31  (95% CI: 0.10–0.91)  p=0.03  G/A  HR=1.25  (95% CI: 0.51–3.07)  p=0.62 |  | G/G  HR=0.15  (95% CI: 0.04–0.57)  p<0.01  G/A  HR=0.63  (95% CI: 0.22–1.76)  p=0.38 |  |  |
| Suh et al.,  2006 [81] | 51 | aCRC | mFOLFOX-4 | XRCC1- Gln399Arg  *c.1196A>G* | rs25487 | tumor tissue | polymorphism | G/G (31)  G/A (16)  A/A (4) |  |  | 30.0 mo vs  16.5 mo vs  12.8 mo  p=0.02 |  |  |
| Lamas et al., 2011 [28] | 72 | aCRC | FUOX | XRCC1- Gln399Arg  *c.1196A>G* | rs25487 | blood | polymorphism | A/A -  G/A –  G/G - | 6 mo vs  10 mo vs  12 mo  p=0.67 |  |  |  |  |
| Zaanan et al., 2014 [30] | 207 | stage III | FOLFOX-4 or FOLFOX-6 | XRCC1- Gln399Arg  *c.1196A>G* | rs25487 | tumor tissue | polymorphism | **G/G** (**94) + G/A (80)**  A/A (33) |  | HR=1.61  (95% CI:  0.82-3.12)  p=0.16 |  |  |  |
| Stoehlmacher et al.,  2004 [31] | 105 | rCRC | FUOX | XRCC1- Gln399Arg  *c.1196A>G* | rs25487 | blood | polymorphism | **G/G (44)**  G/A (51)  A/A (10) | G/A  RR=0.95  (95% CI: 0.60-1.51)  A/A  RR=0.99  (95% CI:  0.47-2.09)  p=0.97 |  | G/A  RR=1.07  (95% CI:  0.63-1.80)  A/A  RR=1.58  (95% CI:  0.71-3.55)  p=0.50 |  |  |
| Liang et al.,  2010 [32] | 113 | mCRC | mFOLFOX-4 or CAPOX | XRCC1- Gln399Arg  *c.1196A>G* | rs25487 | blood | polymorphism | **A/A (61)**  A/G (39)  G/G (13) |  |  |  |  | A/G  HR=1.09  (95% CI:  0.57-2.08)  p=0.80  G/G  HR=1.31  (95% CI:  0.53-3.25)  p=0.57 |
| Gan et al.,  2012 [59] | 289 | aCRC | FOLFOX | XRCC1- Gln399Arg  *c.1196A>G* | rs25487 | blood | polymorphism | **G/G (149)** G/A (88) A/A (51) |  |  | G/A  HR=0.85  (95% CI:  0.51-1.23) A/A  HR=0.66  (95% CI:  0.36-0.95) |  |  |
| Chua et al.,  2009 [39] | 115 | mCRC | FOLFOX | XRCC1- Gln399Arg  *c.1196A>G* | rs25487 | tumor tissue | polymorphism | **G/G (39)**  A/G (61)  A/A (15) | A/G  HR=0.57  (95% CI:  0.28-1.19)  p=0.10 A/A  HR=1.01  (95% CI:  0.39-2.60)  p=1.0 A/G + A/A HR=0.66  (95% CI:  0.34-1.28)  p=0.20 |  | A/G  HR=0.92  (95% CI:  0.58- 1.45)  p=0.70  A/A  HR=0.52  (95% CI:  0.24-1.14)  p=0.10 A/G + A/A  HR=0.83  (95% CI:  0.53-1.29)  p=0.40 |  |  |
| Martinez-Balibrea et al.,  2008 [34] | 47 | mCRC | CAPOX | XRCC1- Gln399Arg  *c.1196A>G* | rs25487 | blood | polymorphism | **G/G (19)**  G/A (19)  A/A (9) | G/A  HR=0.82  (95% CI:  0.39–1.71) A/A  HR=0.65  (95% CI:  0.25–1.66)  p=0.64 |  |  |  |  |
| Martinez-Balibrea et al.,  2008 [34] | 48 | mCRC | FUOX | XRCC1- Gln399Arg  *c.1196A>G* | rs25487 | blood | polymorphism | **G/G (21)**  G/A (20)  A/A (7) | G/A  HR=0.85  (95% CI:  0.42–1.73) A/A  HR=0.96  (95% CI:  0.31–3.00)  p=0.90 |  |  |  |  |
| Huang et al.,  2011 [25] | 157 | mCRC | FOLFOX-4 | ERCC1-Asn118=  *c.354T>C*  and  XRCC1- Gln399Arg  *c.1196A>G* | rs11615 and rs25487 | blood | polymorphism | 2 favorable genotypes  (*ERCC1* C/C and *XRCC1* G/G) - vs  ≤1 favorable genotype - |  |  | 25 mo vs  16.5 mo  p<0.01 |  |  |
| Liang et al.,  2010 [32] | 113 | mCRC | Modified FOLFOX-4 or CAPOX | ERCC1-Asn118=  *c.354T>C*  and  XRCC1- Gln399Arg  *c.1196A>G* | rs11615 and rs25487 | blood | polymorphism | **2 favorable genotypes**  **(*XRCC1* A/A and *ERCC1* C/C) 38** vs  1 favorable genotype 40 vs  0 favorable genotype 35 |  |  |  |  | 1 favorable genotype  HR=2.25  (95% CI:  1.38-3.67)  P<0.01  0 favorable genotype  HR=2.60  (95% CI:  1.56-4.31)  p<0.01 |
| Zaanan et al.,  2014 [30] | 210 | stage III | FOLFOX-4 or FOLFOX-6 | ERCC1-Asn118=  *c.354T>C*  and  XRCC1- Gln399Arg  *c.1196A>G* | rs11615 and rs25487 | tumor tissue | polymorphism | **≥ 1 favorable genotype**  **(*ERCC1* C/C and/or *XRCC1* G/G + G/A) 179** vs  0 favorable genotype 21 |  | HR=2.42  (95% CI:  1.16-5.03)  p=0.02 |  | HR=2.03  (95% CI:  0.96-4.28)  p=0.06 |  |

Abbreviations: aCRC, advanced colorectal cancer; CAPOX, capecitabine and oxaliplatin; CRC, colorectal cancer; CI, confidence interval; FOLFOX, 5-fluorouracil, leucovorin and oxaliplatin; FUOX, 5-fluorouracil and oxaliplatin; HR, hazard ratio; mo, months; mCRC, metastatic colorectal cancer; OS, overall survival; PFS, progression-free survival; rCRC, refractory colorectal cancer. *Reference group in bold.

Table S8. Overview of studies on the association between biomarkers in the HR pathway and treatment outcome of oxaliplatin-based chemotherapy in CRC patients

|  |  |  |  |  |  |  |  |  | *Univariate analysis* | *Multivariate analysis* |
| --- | --- | --- | --- | --- | --- | --- | --- | --- | --- | --- |
| *Author,*  *year* | ***N*** | ***CRC type*** | ***Treatment*** | ***Biomarker*** | ***rs number*** | ***Type of sample*** | ***Type of assay*** | ***Reference*/comparator (n)*** | ***PFS*** | ***PFS*** |
| Martinez-Balibrea et al., 2008 [34] | 47 | mCRC | CAPOX | XRCC3- Thr241Met  *c.722C>T* | rs861539 | blood | polymorphism | **C/C (23)**  C/T (18)  T/T (6) | C/T  HR=0.91  (95% CI:  0.43–1.9) T/T  HR=1.8  (95% CI:  0.66–4.95)  p=0.48 |  |
| Martinez-Balibrea et al., 2008 [34] | 48 | mCRC | FUOX | XRCC3- Thr241Met  *c.722C>T* | rs861539 | blood | polymorphism | **C/C (18)**  C/T (20)  T/T (10) | C/T  HR=1.22  (95% CI:  0.58–2.6) T/T  HR=1.33  (95% CI:  0.54–3.29)  p=0.80 |  |
| Ruzzo et al.,  2007 [40] | 165 | mCRC | FOLFOX-4 | XRCC3- Thr241Met  *c.722C>T* | rs861539 | blood | polymorphism | **T/T (31)**  C/T (71)  C/C (63) | C/T  HR=1.41  (95% CI:  0.89-2.24)  p=0.12  C/C  HR=0.87  (95% CI:  0.54-1.38)  p=0.54 | C/T  HR=1.67  (95% CI:  0.96-2.89)  p=0.07 C/C  HR=0.99  (95% CI:  0.57-1.71)  p=0.96 |
| Ihara et al.,  2016 [82] | 78 | mCRC or rCRC | mFOLFOX or CAPOX ±bevacizumab | MRE11 | n.a. | tumor tissue | protein expression | Positive expression (48)  Negative expression (30) | 11.3 mo vs  11.8 mo  p=0.50 |  |
| Ihara et al.,  2016 [82] | 78 | mCRC or rCRC | mFOLFOX or CAPOX ±bevacizumab | RAD51 | n.a. | tumor tissue | protein expression | **Positive expression (40)**  Negative expression (38) | 9.7 mo vs  13.5 mo  p=0.04 | HR 0.80  (95% CI:  0.35-1.83)  p=0.60 |
| Ihara et al.,  2016 [82] | 78 | mCRC or rCRC | mFOLFOX or CAPOX ±bevacizumab | MRE11 +  RAD51 | n.a. | tumor tissue | protein expression | MREE1 and/or RAD51:  **Positive expression** **47**  Negative expression 31 | 10.1 mo vs  13.2 mo  p=0.02 | HR 1.39  (95%CI:  0.58-3.34)  p=0.50 |
| Moutinho et al., 2014 [79]  discovery cohort | 131 | stage IV | FUOX–based chemotherapy | *SRBC* | n.a. | tumor tissue | DNA methylation status | **Unmethylated (92)**  Methylated (39) | HR=1.83  (95% CI:  1.15-2.92)  p= 0.01 |  |
| Moutinho et al., 2014 [79]  validation cohort | 58 | stage IV | FUOX–based chemotherapy | *SRBC* | n.a. | tumor tissue | DNA methylation status | **Unmethylated (44)**  Methylated (14) | HR=1.90  (95% CI:  1.01-3.60)  p= 0.05 |  |

Abbreviations: CAPOX, capecitabine and oxaliplatin; CRC, colorectal cancer; CI, confidence interval; DFS, disease-free survival; FOLFOX, 5-fluorouracil, leucovorin and oxaliplatin; FUOX, 5-fluorouracil and oxaliplatin; HR, hazard ratio; mo, months; mCRC, metastatic colorectal cancer; OS, overall survival; PFS, progression-free survival; rCRC, refractory colorectal cancer. *Reference group in bold.

Table S9. Overview of studies on the association between biomarkers in the MMR pathway and treatment outcome of oxaliplatin-based chemotherapy in CRC patients

|  |  |  |  |  |  |  |  | *Univariate analysis* | | | *Multivariate analysis* | |
| --- | --- | --- | --- | --- | --- | --- | --- | --- | --- | --- | --- | --- |
| *Author,*  *year* | *n* | *CRC type* | *Treatment* | *Biomarker* | *Type of sample* | *Type of assay* | *Comparator*/reference (n)* | *PFS* | *DFS* | *OS* | *DFS* | *OS* |
| Kim et al., 2010 [70] | 115 | stage II-IV | FOLFOX | MMR status | tumor tissue | protein expression | **pMMR (104)**  dMMR (11) |  | HR=0.69 (0.24–1.97) p=0.49 | HR=1.31  (0.17–9.98)  p=0.79 |  |  |
| Sfakianaki et al.,  2019 [46] | 235 | stage II-III | FOLFOX or CAPOX | MMR status | tumor tissue | polymorphism | **dMMR (35)**  pMMR (200) |  | HR=1.72  (95% CI:  1.29-3.51)  p=0.03 | HR=1.38  (95% CI:  1.04-2.71)  p=0.04 | HR=1.78  (95% CI:  1.34-3.01)  p<0.01 | HR=1.58  (95% CI:  1.24-3.00)  p=0.02 |
| Gallois et al., 2018 [69] | 1867 | stage III | FOLFOX4 ± cetuximab | MMR status | tumor tissue | polymorphism | **dMMR (172)**  pMMR (1560) |  |  |  |  | HR=1.80  (95% CI: 1.16–2.81) p<0.01 |

Abbreviations: aCRC, advanced colorectal cancer; CAPOX, capecitabine and oxaliplatin; CI, confidence interval; CRC, colorectal cancer; DFS, disease-free survival; dMMR, deficient mismatch repair; FOLFOX, 5-fluorouracil, leucovorin and oxaliplatin; FUOX, 5-fluorouracil and oxaliplatin; HR, hazard ratio; MMR, mismatch repair; mo, months; n.s., not specified; OS, overall survival; PFS, progression-free survival; pMMR, proficient mismatch repair. *Reference group in bold.

Table S10. Overview of studies on the association between biomarkers in DNA damage response and DNA synthesis and treatment outcome of oxaliplatin-based chemotherapy in CRC patients

|  |  |  |  |  |  |  |  |  | *Univariate analysis* | | *Multivariate analysis* | |
| --- | --- | --- | --- | --- | --- | --- | --- | --- | --- | --- | --- | --- |
| *Author,*  *year* | *N* | *CRC type* | *Treatment* | *Biomarker* | *rs number* | *Type of sample* | *Type of assay* | *Reference*/comparator (n)* | *PFS* | *OS* | *PFS* | *OS* |
| Sundar et al., 2018. [74] | 121 | mCRC | CAPOX± bevacizumab or FOLFOX± bevacizumab/cetuximab | ATM | n.a. | tumor tissue | protein expression | **loss (9)**  proficient (113) |  | HR=2.52  (95% CI:  1.00-6.37)  p=0.05 |  |  |
| Kweekel et al., 2009 [65] | 91 | aCRC | CAPOX | ATM-Asp1853Asn  *c.5557G>A* | rs1801516 | blood | polymorphism | **G/G (63)**  G/A (24)  A/A (4) |  | No difference | G/A HR=0.72 (95% CI: 0.43-1.21) A/A  HR=4.25 (95% CI: 1.45-12.44)  p<0.01 |  |
| Okazaki et al., 2017 [76] | 218 | mCRC | Oxaliplatin-based chemotherapy | *HIC1*  *Tandem repeat D17S5 loci* | n.a. | blood | polymorphism | **S/S (≤4TRs in both alleles) 179 + S/L (≤4TRs in one allele) 19**  L/L(≥5TRs in both alleles) 20 | HR=1.93  (95% CI: 1.11–3.35) p=0.01 | HR=1.25  (95% CI:  0.74, 2.10)  p=0.40 | HR=2.00  (95% CI: 1.13–3.54) p=0.02 | HR=1.20 (95% CI: 0.71, 2.04)  p=0.50 |
| Suenaga et al., 2018 [78] | 143 | mCRC | FOLFOX ± bevacizumab | *PIN1*  *NC_000019.9:g.9945179G>C* | rs2233678 | blood | polymorphism | **G/G (129)**  G/C (13) + C/C (1) | HR=3.24 (95% CI: 1.60- 6.54) p<0.01 | HR=2.38  (95% CI:  1.32- 4.30)  p<0.01 | HR= 2.67 (95% CI:  1.28-5.57)  p<0.01 | HR=1.91 (95%CI: 1.02-3.59)  p=0.04 |
| Suenaga et al., 2018 [78] | 70 | mCRC | FOLFOX or CAPOX + bevacizumab | *PIN1*  *NC_000019.9:g.9945179G>C* | rs2233678 | blood | polymorphism | **G/G (64)**  G/C (6) | HR=1.11 (95% CI: 0.43-2.82)  p=0.83 | HR=2.43  (95% CI:  0.83-7.15)  p=0.09 | HR=1.15 (95% CI:  0.44-2.98)  p=0.78 | HR=3.01 (95% CI: 0.98-9.20)  p=0.05 |
| Park et al.,  2010 [80] | 88 | mCRC | CAPOX or mFOLFOX4 | *MGMT*  *-535G>T* | rs1625649 | tumor tissue | polymorphism | **G/G (39) + G/T (39)**  T/T (10) | HR=2.65 (95% CI: 1.10–6.39) p=0.03 | HR=2.09  (95% CI:  0.59–7.47)  p=0.26 | HR=3.14  (95% CI: 1.42–6.91) p<0.01 | HR=2.06  (95% CI: 0.74–5.75)  p=0.17 |

Abbreviations: *ATM, ataxia-telangiectasia mutated*; CAPOX, capecitabine and oxaliplatin; CRC, colorectal cancer; CI, confidence interval; FOLFOX, 5-fluorouracil, leucovorin and oxaliplatin; FUOX, 5-fluorouracil and oxaliplatin; HIC1, hypermethylated in cancer 1; HR, hazard ratio; *MGMT, Human O6-alkylguanine-DNA alkyltransferase*; mo, months; mCRC, metastatic colorectal cancer; OS, overall survival; PFS, progression-free survival; *PIN1, peptidyl-prolyl cis/trans isomerase NIMA-interacting 1*; TR, tandem repeat. *Reference group in bold.
